# Identification of ARHGEF38, NETO2, GOLM1, and SAPCD2 Associated With Prostate Cancer Progression by Bioinformatic Analysis and Experimental Validation

**DOI:** 10.3389/fcell.2021.718638

**Published:** 2021-09-01

**Authors:** Zhuolun Sun, Yunhua Mao, Xu Zhang, Shuo Lu, Hua Wang, Chi Zhang, Chutian Xiao, Yinghao Cao, Yunhao Qing, Yu Wang, Ke Li

**Affiliations:** ^1^Department of Urology, Third Affiliated Hospital of Sun Yat-sen University, Guangzhou, China; ^2^Department of Gynecology, Third Affiliated Hospital of Sun Yat-sen University, Guangzhou, China; ^3^Department of Gastrointestinal Surgery, Union Hospital, Tongji Medical College, Huazhong University of Science and Technology, Wuhan, China

**Keywords:** prostate cancer, prognostic signature, TCGA, GEO, WGCNA

## Abstract

Prostate cancer (PCa) represents one of the most prevalent types of cancers and is a large health burden for men. The pathogenic mechanisms of PCa still need further investigation. The aim of this study was to construct an effective signature to predict the prognosis of PCa patients and identify the biofunctions of signature-related genes. First, we screened differentially expressed genes (DEGs) between PCa and normal control tissues in The Cancer Genome Atlas (TCGA) and GSE46602 datasets, and we performed weighted gene co-expression network analysis (WGCNA) to determine gene modules correlated with tumors. In total, 124 differentially co-expressed genes were retained. Additionally, five genes (ARHGEF38, NETO2, PRSS21, GOLM1, and SAPCD2) were identified to develop the prognostic signature based on TCGA dataset. The five-gene risk score was verified as an independent prognostic indicator through multivariate Cox regression analyses. The expression of the five genes involved in the signature was detected in the Gene Expression Omnibus (GEO), Gene Expression Profiling Interactive Analysis (GEPIA), and Oncomine databases. In addition, we utilized DiseaseMeth 2.0 and MEXPRESS for further analysis and found that abnormal methylation patterns may be a potential mechanism for these five DEGs in PCa. Finally, we observed that these genes, except PRSS21, were highly expressed in tumor samples and PCa cells. Functional experiments revealed that silencing ARHGEF38, NETO2, GOLM1, and SAPCD2 suppressed the proliferation, migration, and invasiveness of PCa cells. In summary, this prognostic signature had significant clinical significance for treatment planning and prognostic evaluation of patients with PCa. Thus, ARHGEF38, NETO2, GOLM1, and SAPCD2 may serve as oncogenes in PCa.

## Introduction

Prostate cancer (PCa) is a common malignant neoplasm among men in the urogenital system that not only impairs the patients’ quality of life but also simultaneously causes financial burdens for society and the family (Saad, 2019). PCa accounts for nearly one in five newly diagnosed cancers and has become the second most prevalent malignancy. Moreover, the estimated deaths caused by PCa rank second (33,330 deaths) in American men, ranking only after gastric cancer (Siegel et al., 2020). The etiology and early events in the progression of PCa remain unclear, and a combination of factors likely contributes to its development (Rui et al., 2019). Several therapies are currently used for the treatment of PCa, including active surveillance, radical prostatectomy, conventional chemotherapy, external beam radiation, targeted therapy, and hormonal therapy (Skolarus et al., 2014). With the improvement of therapeutic strategies, the incidence of patient mortality has decreased. Unfortunately, previous studies have reported that up to 25% of men who undergo curatively intended treatment will experience biochemical recurrence (BCR; Brockman et al., 2015). Patients with BCR then have a risk of metastasis and cancer death (Shao et al., 2020).

At present, PCa can be diagnosed in the early stage by the detection of prostate-specific antigen (PSA), which is a routinely and extensively utilized circulating marker (Grossman et al., 2018). However, PSA testing presents high false-positive rates that contribute to overdiagnosis, unnecessary biopsies, and overtreatment because it is also associated with other benign diseases, such as benign prostatic hyperplasia (BPH) or prostatitis (Loeb et al., 2014; Ferrer-Batallé et al., 2017). Additionally, not all PCa cases give rise to an elevated serum PSA concentration. To further aid the decision-making process for clinicians, the ratio-free PSA/total PSA, PSA density, four-kallikrein panel, and prostate health index have been introduced (Kohaar et al., 2019). However, the prediction accuracy of PCa prognostic markers still needs to be improved. Currently, the identification of effective biomarkers for the prediction and prognosis of PCa is still one of the utmost challenges in clinical practice.

The advancement of high-throughput next-generation sequencing technologies and bioinformatics has revolutionized gene expression research and our understanding of cancer (Zeng et al., 2013). Transcriptome data analysis is an efficient way to identify differentially expressed genes (DEGs) at the whole genome level, which provides a method to explore the underlying molecular mechanisms of gene expression regulation and to investigate the quantitative change in expression profiles between the control and treatment groups (To, 2000). Furthermore, weighted gene correlation network analysis (WGCNA) has been demonstrated as an efficient systems biology approach designed for constructing a co-expression network among the identified genes (Langfelder and Horvath, 2008). This method clusters highly relevant genes into a module and relates them to clinical characteristics, which may be helpful in screening tumor markers to improve the level of clinical early diagnosis and ultimately benefit improved therapeutic effects. To date, many studies have considered the expression of genes individually and ignored the underlying high degree of interconnection among genes.

In the present study, we applied the above two methods to combine the WGCNA findings with DEG analysis to identify differentially co-expressed genes. Thus, our study not only focused on DEGs but also fully considered the internal relationships among genes. We then constructed a prognostic signature as an independent factor in PCa patients using integrated bioinformatics methods based on The Cancer Genome Atlas (TCGA) database. In addition, we used several public databases to verify the stability and reliability of our results. Finally, functional analyses (*in vitro*) were further performed to confirm the role of these selected genes in PCa.

## Materials and Methods

### Data Source

In the present study, the corresponding raw transcriptome count data and clinical information of patients with PCa were obtained from TCGA database^1^. This dataset is comprised of 489 PCa and 51 non-cancerous prostate samples as well as RNA-seq count data for 19,648 genes. For the convenience of downstream analysis, all probe identifiers were converted to Ensembl gene IDs using the human genome sequence (GRCh38/hg38)^2^ and annotation GTF file (GENCODE version 26). Annotation probes were not removed, and analysis of gene expression was performed only on genes exceeding an average of >1 counts per million (CPM). Finally, a total of 14,440 genes with read per kilobase million (RPKM) values were utilized for further analysis after filtering.

Subsequently, the expression profile of GSE46602 submitted by Mortensen et al. (2015) was acquired from the Gene Expression Omnibus (GEO) database using the keyword “prostate cancer” in NCBI^3^. The GSE46602 dataset, which was generated using the GPL570 platform (HG-U133_Plus_2) Affymetrix Human Genome U133 Plus 2.0 Array, includes 36 PCa samples and 14 normal prostate samples. Probe sets corresponding to the Arabidopsis Genome Initiative (AGI) gene identifiers were located based on the probe annotation file from GEO, and the average value was used as the reference if multiple probes targeted the same gene. Finally, 21,654 genes were selected for subsequent analysis.

### Analysis of DEGs Between PCa and Normal Samples

Publicly available microarray data were subjected to background correction and normalization as well as differential expression analysis using the “limma” R package (Ritchie et al., 2015). The “limma” package was utilized to identify the DEGs between PCa tissues and normal tissues in both TCGA and GSE46602 datasets. The *P* values were adjusted for multiple testing correction by false discovery rate (FDR; Benjamini and Hochberg, 1995). DEGs were recruited with | log2FC| value > 1 and FDR < 0.05.

### WGCNA and Clinically Significant Module Identification

Weighted gene co-expression network analysis was applied to identify interesting gene modules highly connected with other genes (Langfelder and Horvath, 2008). To determine potential highly co-expressed clusters of genes, the gene profiles of TCGA and GSE46602 datasets were utilized to construct co-expression networks with the “WGCNA” package. First, the samples were subjected to cluster analysis through the “hclust” function to verify and eliminate outliers. Second, an adjacency matrix was generated by analyzing the *P*earson correlation between each pair of extracted genes. Third, a soft-thresholding parameter power value (β) that could emphasize the potent association of genes while penalizing the low associations to ensure the scale-free network was constructed (Botía et al., 2017). Moreover, hierarchical cluster analysis was performed to identify modules with genes of similar expression profiles according to the TOM-based dissimilarity (1-TOM) with over 50 genes for a dendrogram. A cutoff (<0.25) was then selected to merge similar modules to make the results more reliable. Network construction and consensus module detection were performed by using the “DynamicTreeCut” algorithm.

The correlations between modules and PCa were calculated using the module–trait relationships of WGCNA. In addition, we regarded the log10 transformation of the *P* value (lgP) for the association of gene expression and clinical condition as the gene significance (GS). The module eigengene (ME; representative of the gene expression profiles from a module) was considered the first principal component of a given module. Ultimately, the clinical significance module was determined by calculating the relationship between clinical traits and MEs. The module that exhibited the highest Pearson’s correlation coefficient was considered a candidate relevant to clinical traits and was selected for subsequent study.

A Venn diagram was constructed to identify the overlapping genes between DEGs (extracted from differential expression analysis) and co-expressed genes (extracted from WGCNA), which was drawn by the “Venn Diagram” package. To further understand the biofunction of overlapping genes, we employed Gene Ontology (GO) enrichment and Kyoto Encyclopedia of Genes and Genomes (KEGG) pathway analyses using the “clusterProfiler” package. GO annotation included biological process (BP), cellular component (CC), and molecular function (MF) terms. A *P* value cutoff of 0.05 was used for significant enrichment.

### Construction and Validation of the Prognostic Signature

Univariate Cox analysis was performed to evaluate the correlation between 124 genes and overall survival (OS) for PCa patients with the “survival” package based on TCGA dataset. Hazard ratios (HRs) were used to confirm risk (HR > 1) and protective (HR < 1) genes. Based on the results of univariate analysis, only the top 10 candidate genes highly associated with OS were retained for subsequent analysis. Multivariate Cox regression analysis was applied to identify the final hub genes and their estimated regression coefficients (β) with the lowest Akaike information criterion (AIC) value. The risk score formula for each patient was constructed with the following formula: risk score = $\sum_{i=1}^{n}C\,o\,e\,f\,\left(i\right)\times x\,\left(i\right)$; where *Coef*(*i*) indicates the regression coefficient for each gene, and *x*(*i*) indicates the gene expression level. All PCa patients were assigned into high- or low-risk groups based on the median risk score. Kaplan–Meier survival curves were used to estimate the discriminative ability of patient prognosis, and receiver operating characteristic (ROC) curves were employed to evaluate the prognostic power of the signature. The independent prognostic factors were determined by Cox regression analysis. Moreover, the GSE46602 dataset was used as an external validation dataset.

### Verification of the Signature-Related Genes

To further confirm the reliability of the signature-related genes, we compared the expression patterns between tumors and normal tissues in TCGA, GEO (GSE46602 and GSE6032), and Oncomine databases^4^ (Rhodes et al., 2004). The cBioPortal Cancer Genomics Portal database^5^ (Cerami et al., 2012), an online open-access resource for exploring a multidimensional cancer genomics dataset, was utilized to investigate the genetic alterations of the genes involved in the signature.

Additionally, to further explore the potential ability of each prognostic gene in the signature, we evaluated the association between OS and signature-related genes expressed in PCa patients through the Gene Expression Profiling Interactive Analysis (GEPIA) database^6^ (Tang et al., 2017), and log-rank tests were used to measure statistical significance. The area under the curve values were calculated to measure the prediction performances of the single selected genes.

The human disease methylation database (DiseaseMeth version 2.0)^7^ is a website focusing on collecting methylation data from tumor and adjacent normal tissues (Xiong et al., 2017). We employed this online tool to investigate methylation levels of the signature-related genes between PCa and normal tissues. MEXPRESS^8^ is a data visualization tool for gene expression, DNA methylation, and clinical information from TCGA. Using MEXPRESS, we not only investigated the available DNA methylation data at individual cytosine-phosphate-guanines (CpGs) related to their precise genomic location but also explored the relationships between the DNA methylation data and gene expression and several clinical parameters (Koch et al., 2019).

### Tissue Samples

A total of 25 PCa tissues and 25 BPH tissues were acquired from patients who underwent surgery at the Third Affiliated Hospital of Sun Yat-sen University (Guangzhou, China). None of the PCa patients had received hormonal treatment, chemotherapy, or preoperative radiotherapy. All collected tissues were stored at −80°C until further quantitative real-time PCR (qRT–PCR) analysis. Written informed consent was obtained from all subjects, and the present study was approved by the Ethical Committee at the Third Affiliated Hospital of Sun Yat-sen University.

### Immunohistochemistry

The resected fresh tissues were immediately fixed with 10% formalin and embedded in paraffin. The paraffin-embedded tissues were sectioned at a 4 mm thickness and sequentially arranged on glass slides. Briefly, the tissue sections were routinely deparaffinized with xylene and rehydrated in graded ethanol followed by incubation with 0.3% hydrogen peroxide for 10 min to block endogenous peroxidase activity. Antigen retrieval was achieved in 0.01 M citrate buffer (pH = 6.0) for 15 min at 100°C using a microwave oven followed by incubation overnight at 4°C with the following primary antibodies: rabbit anti-ARHGEF38 (1:100, ab122345, Abcam), rabbit anti-NETO2 (1:20, NBP-84624, Novus), rabbit anti-PRSS21 (1:20, Ab251695, Abcam), rabbit anti-GOLM1 (GOLPH2) (1:500, ab109628, Abcam), and rabbit anti-SAPCD2 (1:1000, NBP1–91740, Novus). After being washed three times in Tris-buffered saline with Tween (TBST), tissue sections were incubated with a secondary antibody (1:2000, ab6271, Abcam) for 30 min at 37°C. Finally, the above sections were counterstained with hematoxylin, dehydrated, cleared, and coverslipped for light microscopic examination.

### qRT–PCR

Total RNA was extracted with TRIzol reagent (Invitrogen, Grand Island, NY, United States) and reverse transcribed into cDNA using the PrimeScript RT reagent Kit (TaKaRa, Japan) following the manufacturer’s protocols. qRT–PCR was performed with a SYBR Green I Master Kit (Roche) on a LightCycler^®^ 480 System (Roche). PCR conditions were forty-five cycles at 95°C for 5 min, followed by 95°C for 10 s, 60°C for 10 s, and 72°C for 10 s. The relative mRNA levels were normalized against β-actin based on the 2^–ΔΔ*Ct*^ method. The sequences of the qRT–PCR primers are listed in Supplementary Table 1.

### Cell Culture and Cell Transfection

One normal prostate epithelial cell line (RWPE-1) and two PCa cell lines (22Rv1 and PC-3) were obtained from The American Type Culture Collection (ATCC, VA, United States). PCa cells were cultured in Dulbecco’s modified Eagle’s medium (DMEM, Thermo Fisher Scientific, Waltham, MA, United States) supplemented with 10% fetal bovine serum (FBS), while RWPE-1 cells were cultured in supplemented keratinocyte serum-free medium (K-SFM) (Thermo Fisher Scientific). All cell lines were grown in a 5% CO_2_-humidified incubator at 37°C.

The mRNA expression levels of ARHGEF38, NETO2, GOLM1, and SAPCD2 were knocked down using small interfering RNAs (siRNAs). Si-ARHGEF38, si-NETO2, si-GOLM1, si-SAPCD2, and si-NC (negative control) were commercially constructed by GenePharma (Shanghai, China). The sequences of these siRNAs are listed in Supplementary Table 2. After reaching a cell density of 60–70%, 22Rv1 and PC-3 cells were transfected with these siRNAs using Lipofectamine 3000 according to the manufacturer’s instructions (Invitrogen, Thermo Fisher Scientific, Waltham, MA, United States). The assays and further experiments were performed 48 h after transfection.

### Cell Proliferation Assay

Cell proliferation was determined by dimethylthiazolyl diphenyltetrazolium bromide (MTT) kit (BioFroxx, Einhausen, Germany) according to the manufacturer’s instructions. Briefly, after 48 h of transfection, 22Rv1 and PC-3 cells were seeded into 96-well plates (5,000 cells per well in 200 μL of complete culture medium). Following incubation for 24, 48, 72, 96, and 120 h, 10 μL of MTT reagent was added to each well followed by incubation for 4 h. The formed formazan crystals were dissolved in 200 μL of dimethyl sulfoxide (DMSO), and the optical density (OD) value of each well was then measured at 490 nm.

### Transwell Assays

Cell migration and invasion capabilities were examined using 24-well Transwell inserts with 8-μm pores (EMD Millipore Inc., MA, United States). Briefly, transfected cells (3 × 10^4^ suspended in 200 μL of serum-free medium) were seeded into the upper chamber of a Transwell insert precoated without (migration) or with (invasion) Matrigel (BD Bioscience, United States). The lower chamber was filled with medium containing 10% FBS as a chemoattractant. After culturing for 48 h, cells in the upper chamber were gently removed, and cells that migrated or invaded through the chambers were fixed, stained, and visualized by microscopy (Nikon). Five visual fields were randomly selected for each chamber, and experiments were repeated independently at least three times.

### Statistical Analysis

All data were analyzed by R software 4.0.1 (R Core Team), GraphPad Prism v7.0 (GraphPad, San Diego, CA, United States), and SPSS v23.0 (SPSS, Chicago, IL, United States). Each experiment was repeated independently three times. Data are expressed as the mean ± standard deviation (SD), and *P* < 0.05 was considered statistically significant.

## Results

### Identification of DEGs

**FIGURE 1:**
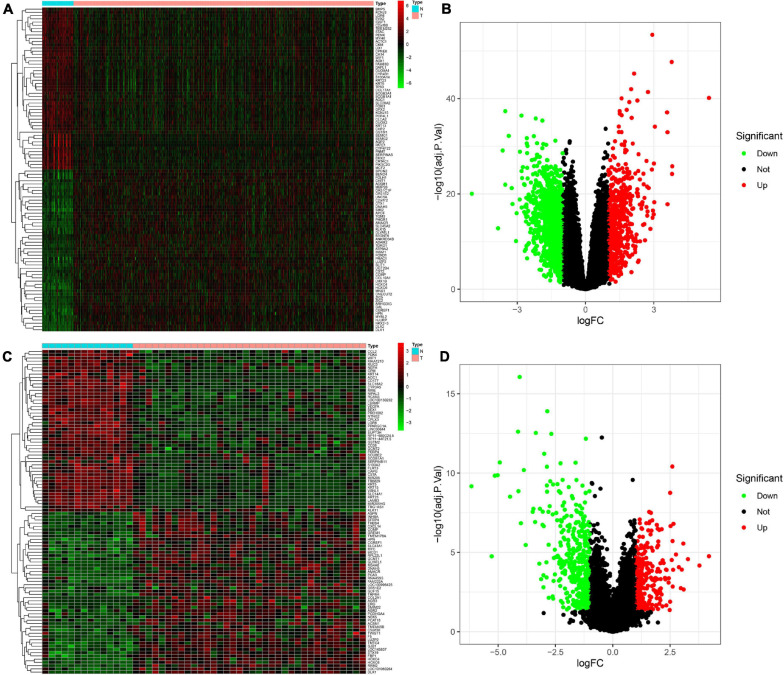
Identification of differentially expressed genes (DEGs) between Prostate cancer (PCa) and normal tissues. **(A,B)** Heatmap **(A)** and volcano plot **(B)** of DEGs in The Cancer Genome Atlas (TCGA) dataset. **(C,D)** Heatmap **(C)** and volcano plot **(D)** of DEGs in the GSE46602 dataset.

The DEGs between PCa and normal prostate samples in TCGA dataset and the GSE46602 dataset were identified using the “limma” package. In total, 1,668 DEGs in TCGA dataset, including 522 upregulated and 1,146 downregulated genes, were determined. The differential genes between the PCa group and the normal control group are presented in a heatmap (Figure 1A) and volcano plot (Figure 1B). The same screening strategy was applied to the GSE46602 dataset, identifying 694 DEGs with 251 upregulated and 446 downregulated genes. The corresponding heatmap and volcano plot are illustrated in Figures 1C,D.

### WGCNA and Key Module Identification

**FIGURE 2:**
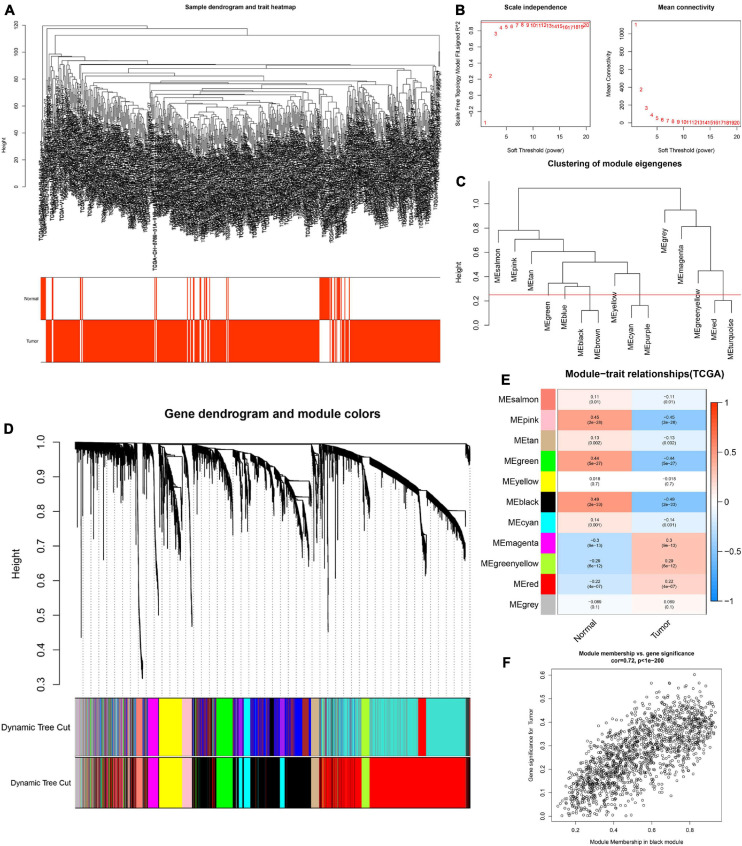
Identification of modules related to the clinical traits in TCGA dataset. **(A)** Clustering dendrograms of samples as well as the clinical features. **(B)** The left panel shows the scale-free fitting indices for various soft-thresholding powers (β). The right panel shows the mean connectivity as a function of the soft-threshold power. **(C)** Clustering of module eigengenes. The red line indicates the cutoff (0.25). **(D)** Cluster dendrogram of co-expression network modules based on the 1-TOM matrix. **(E)** Heatmap of the correlation between module eigengenes and clinical traits of PCa. Each cell contains the corresponding correlation and *P* value. **(F)** Scatter plot of module eigengenes in the black module. Each module represents a cluster of co-related genes and was assigned a unique color.

**FIGURE 3:**
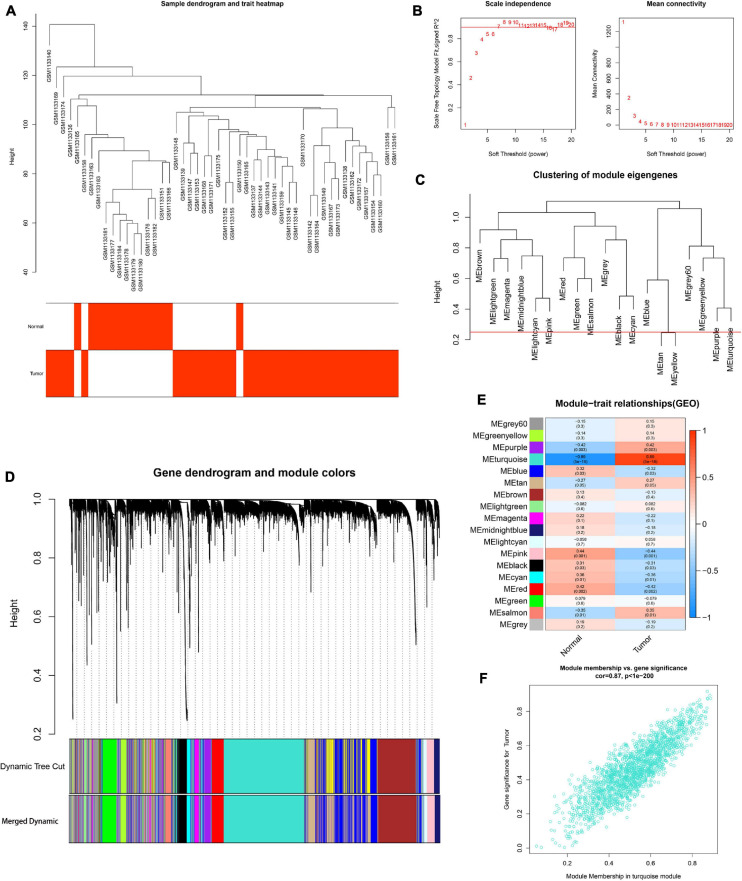
Identification of modules associated with the clinical information in the GSE46602 dataset. **(A)** Clustering dendrograms of samples as well as the clinical features. **(B)** The left panel shows the scale-free fitting indices for various soft-thresholding powers (β). The right panel shows the mean connectivity as a function of the soft-threshold power. **(C)** Clustering of module eigengenes. The red line indicates cutoff (0.25). **(D)** Cluster dendrogram of co-expression network modules based on the 1-TOM matrix. **(E)** Heatmap of the correlation between module eigengenes and clinical traits of PCa. Each cell contains the corresponding correlation and *P* value. **(F)** Scatter plot of module eigengenes in the turquoise module. Each module represents a cluster of co-related genes and was assigned a unique color.

To determine the hub modules most related to PCa patients, co-expression analysis was performed to construct the gene co-expression networks from TCGA dataset and the GSE46602 dataset. The tumor samples of TCGA dataset (Figure 2A) and the GSE46602 dataset (Figure 3A) were clustered with the Pearson correlation method and the average linkage method. No outlier sample was detected or removed. To ensure that the constructed networks were scale-free, we selected the optimal β = 4 (scale-free *R*^2^ = 0.9) (Figure 2B) in TCGA dataset and β = 7 (scale-free *R*^2^ = 0.9) (Figure 3B) in the GSE46602 dataset. By using a cutoff of 0.25 and minimal module size of 50, 15 modules in TCGA (Figures 2C,D) and 18 modules in GSE46602 (Figures 3C,D) were retained for subsequent analyses (gray module indicates no assignment to any cluster).

We then evaluated the relationship between each module and clinical features using the heatmap. After module-trait relationship analysis, the black module (*r* = −0.49, *P* = 2e-33) (Figure 2E) in TCGA and the turquoise module (*r* = 0.86, *P* = 5e-18) (Figure 3E) in GSE46602 had the highest association with PCa. The expression levels of genes in the black module were negatively associated with PCa, while those of the turquoise module were positively associated with PCa. In total, 1,549 and 1,966 co-expressed genes were identified in the black module of TCGA dataset and the turquoise module of the GSE46602 dataset, respectively. In addition, the scatter plots of the blue module (Figure 2F) and the turquoise module (Figure 3F) showed a high correlation between GS and module membership. Thus, the black module of TCGA dataset and the turquoise module of the GSE46602 dataset were regarded as two promising PCa-related modules.

### Identification of Overlapping Genes and Functional Enrichment Analysis

**FIGURE 4:**
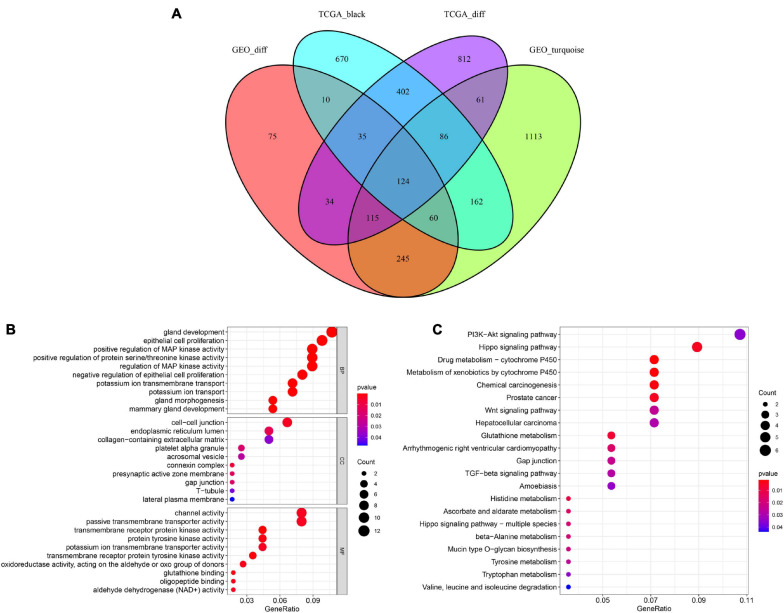
Identification of overlapping genes among DEG lists, co-expression modules, and functional enrichment analysis. **(A)** Venn diagram of overlapping genes among DEG lists and co-expression modules. **(B)** Enriched biological process (BP), cellular component (CC), and molecular function (MF) of the overlapping genes. **(C)** Enriched Kyoto Encyclopedia of Genes and Genomes (KEGG) pathways of the overlapping genes.

Based on the above results, we identified a total of 1,668 DEGs and 1,549 co-expressed genes in TCGA dataset in addition to 694 DEGs and 1,966 co-expressed genes in the GSE46602 dataset. The Venn diagram indicated that there were 124 overlapping genes between the different groups (Figure 4A).

To further investigate the potential molecular mechanisms of the 124 genes in PCa, GO, and KEGG functional annotation analyses were performed. GO analysis suggested that 124 overlapping genes were significantly associated with the following BPs, including gland development, epithelial cell proliferation, and positive regulation of MAP kinase activity (Figure 4B). CC analysis showed that cell-cell junctions, endoplasmic reticulum lumen, and collagen-containing extracellular matrix were the most common classifications. For MF, these genes were mainly associated with channel activity, passive transmembrane transporter activity, and transmembrane receptor protein kinase activity. Moreover, our results revealed that 124 overlapping genes were involved in the PI3K-Akt signaling pathway and Hippo signaling pathway through KEGG enrichment analysis (Figure 4C). These results showed that these 124 overlapping genes are significantly involved in PCa progression.

### Construction and Validation of a Signature to Predict the Prognosis of PCa Patients

To study the prognostic value of 124 overlapping genes in PCa, we performed univariate Cox regression analysis based on TCGA data and identified genes highly correlated with OS. The top 10 genes were as follows: ARHGEF38, LPAR1, NETO2, PRSS21, SLC12A8, GOLM1, GPR160, FAM107A, SAPCD2, and GPX3. Among these 10 genes, ARHGEF38, NETO2, SLC12A8, GOLM1, GPR160, and SAPCD2 were risk genes with HR > 1, while LPAR1, PRSS21, FAM107A, and GPX3 were protective genes with HR < 1. Five genes, including ARHGEF38, NETO2, PRSS21, GOLM1, and SAPCD2, were identified to construct the prediction signature based on the lowest AIC value in the multivariate Cox regression analysis. The risk score was calculated as follows: risk score = (0.2036 × expression level of ARHGEF38) + (−0.4913 × expression level of NETO2) + (−1.5379 × expression level of PRSS21) + (0.0025 × expression level of GOLM1) + (0.1365 × expression level of SAPCD2).

**FIGURE 5:**
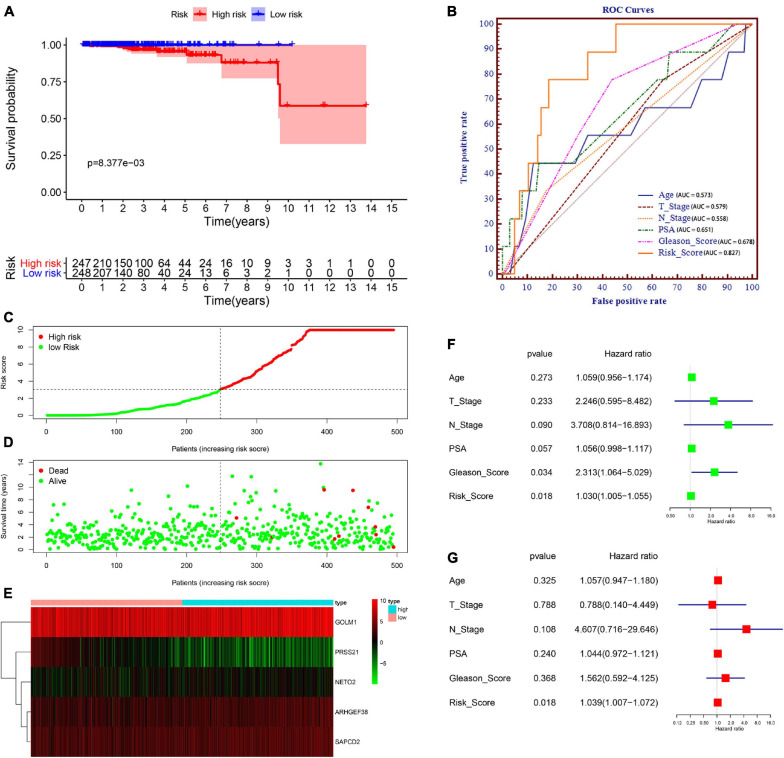
Prognostic signature of PCa patients based on TCGA database. **(A)** Kaplan–Meier survival curves for patients in the high- and low-risk groups. **(B)** Receiver operating characteristic (ROC) curve for survival predictions for the constructed signature in comparison to several clinical parameters, including age, N stage, T stage, prostate-specific antigen (PSA), and Gleason score. **(C)** Distributions of risk scores. **(D)** Distributions of overall survival (OS) status. **(E)** Heatmap of the signature-related gene expression profiles. **(F,G)** Univariate **(F)** and multivariate **(G)** Cox regression analyses verified the independent value of the signature for overall survival.

We then divided PCa patients into high- and low-risk groups based on the median risk scores. Kaplan–Meier analysis revealed that the survival of PCa patients in the high-risk group was significantly shorter than that in the low-risk group (*P* = 8.377e–03) (Figure 5A). To validate the potential diagnostic and recognition effectiveness of the constructed prognostic signature, ROC analysis was applied, and the AUC value was calculated. Furthermore, we also evaluated the AUC values of several clinical parameters, including age, N stage, T stage, PSA value, and total Gleason score. The signature curve showed the greatest AUC value (AUC = 0.827) compared to the age curve (AUC = 0.573), N stage curve (AUC = 0.579), T stage curve (AUC = 0.558), PSA value curve (AUC = 0.651), and total Gleason score curve (AUC = 0.678) (Figure 5B). These results suggested that the constructed prognostic signature may be better than the other clinical parameters and more accurate in differentiating PCa patients. Figure 5C shows the distributions of the prognostic signature-based risk scores. The survival status of PCa patients was marked on the dot plot, and the results showed that the mortality rate of patients increased with the increase in risk score (Figure 5D). Heatmap analysis revealed distinct patterns of gene expression between the groups. For patients with high-risk scores, the expression levels of four risk genes were upregulated, and one protective risk gene was downregulated. In contrast, the expression of prognostic genes presented the opposite patterns in the low-risk group (Figure 5E).

Subsequently, to examine whether the prognostic signature-based risk score is an independent prognostic indicator, Cox regression analyses were performed using the prognostic signature-based risk score and relevant clinical indicators (including age, N stage, T stage, PSA value, and total Gleason score) in TCGA dataset. As a result, the N stage, Gleason score, and risk score were closely correlated with OS in univariate analysis (Figure 5F), and only the risk score was still significantly related to OS in multivariate analysis (Figure 5G). Together, these findings indicated that the signature is an independent prognostic indicator for predicting OS in patients with PCa.

In addition, we used the GSE46602 dataset to validate our signature. Due to the few dead patients in the GSE46602 dataset, we used BCR-free survival to prove its utility. Kaplan–Meier curves demonstrated that patients in the high-risk group had a poorer prognosis than those in the lower-risk group (*P* < 0.05) (Supplementary Figure 1A). The ROC curve showed great classifying efficacy of the constructed signature (AUC = 0.756) (Supplementary Figure 1B).

### Validation of the Expression Pattern, Prognostic Value, and Methylation Level of the Signature-Related Genes

**FIGURE 6:**
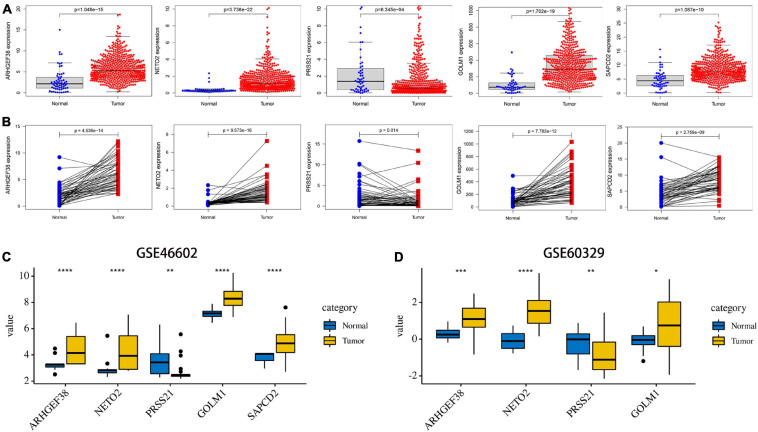
Validation of the expression levels of the five signature-related genes in TCGA and Gene Expression Omnibus (GEO) databases. **(A)** ARHGEF38, NETO2, PRSS21, GOLM1, and SAPCD2 gene expression differences between PCa and normal tissues in TCGA dataset. **(B)** ARHGEF38, NETO2, PRSS21, GOLM1, and SAPCD2 gene expression differences between PCa and paired adjacent non-tumor prostate tissues in TCGA dataset. **(C,D)** ARHGEF38, NETO2, PRSS21, GOLM1, and SAPCD2 gene expression differences between PCa and normal tissues in the GSE46602 dataset **(C)** and the GSE60329 dataset **(D)**. *P < 0.05, **P < 0.01, ***P < 0.001, and ****P < 0.0001.

**FIGURE 7:**
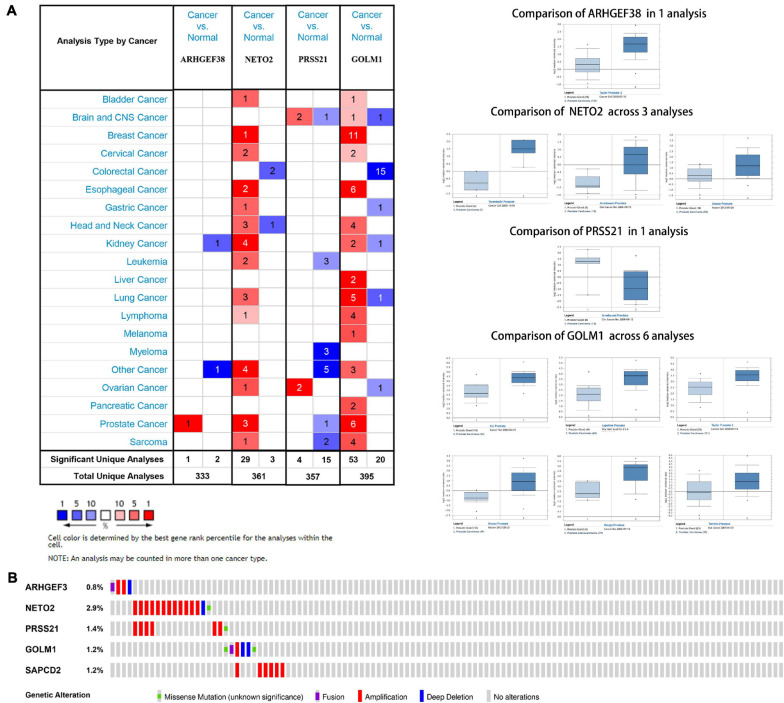
Expression and genetic alterations of the five signature-related genes. **(A)** An overview of the expression levels of five signature-related genes in the Oncomine database. **(B)** Genetic alterations of ARHGEF38, NETO2, PRSS21, GOLM1, and SAPCD2 in TCGA-PCA patients based on the cBioPortal for Cancer Genomics database.

After these signature-related genes were screened out, we then validated the differential expression between tumors and normal tissues. In PCa tissues compared to normal control tissues based on TCGA database, four risk genes (ARHGEF38, NETO2, GOLM1, and SAPCD2) were significantly upregulated, while one protective gene (PRSS21) was downregulated (Figure 6A). In addition, these gene expression patterns agreed with the results described above using TCGA paired data (Figure 6B). The expression patterns of signature-related genes were further confirmed in internal (GSE46602) (Figure 6C) and external (GSE60329) validation datasets (Figure 6D) from the GEO database and in the Oncomine online database (Figure 7A), which were consistent with the above results. Because there was no information available for the SAPCD2 expression profile in the GSE60329 dataset and Oncomine database, we did not further investigate SAPCD2.

The genetic alterations of the five genes were analyzed according to the cBioPortal database. Among these five genes, NETO2 possessed the most frequent genetic alterations (Figure 7B). In addition, we observed that the most common type of alteration was amplification in all genes, except for GOLM1. The abovementioned observations confirmed aberrant expression statuses of the signature-related genes, and genetic alteration, at least in part, may contribute to the aberrant expression of the corresponding genes.

Subsequently, OS analyses of these five genes were performed using the GEPIA database to further investigate the prognostic values of the signature-related genes in PCa patients. Kaplan–Meier analyses suggested that higher expression levels of ARHGEF38 and GOLM1 were significantly associated with worse OS (*P* < 0.05), while no significant difference was found in OS related to the expression levels of NETO2, PRSS21, and SAPCD2 (Supplementary Figure 2A). In addition, we observed that these genes presented a lower AUC value when they were evaluated alone (ARHGEF38, AUC = 0.688; NETO2, AUC = 0.604; PRSS21, AUC = 0.753; GOLM1, AUC = 648; and SAPCD2, AUC = 0.673) (Supplementary Figure 2B) compared to the constructed signature. Notwithstanding, these genes exhibited a competitive performance for survival prediction of PCa.

To further understand the methylation levels of these signature-related genes, the DNA methylation data in PCa were downloaded from the DiseaseMeth database. The results indicated that the methylation levels of ARHGEF38, NETO2, GOLM1, and SAPCD2 (risk genes) were lower in PCa than in normal tissues (Supplementary Figure 3). In contrast, PRSS21 (a protective gene) presented hypermethylation in the disease state. Additionally, MEXPRESS analysis indicated that DNA methylation at multiple sites of these signature-related genes negatively correlated with their expression (Supplementary Figure 4). This potential mechanism may be the main reason for the aberrant expression of these signature-related genes in PCa.

### Expression Levels of Signature-Related Genes in Clinical Patients

**FIGURE 8:**
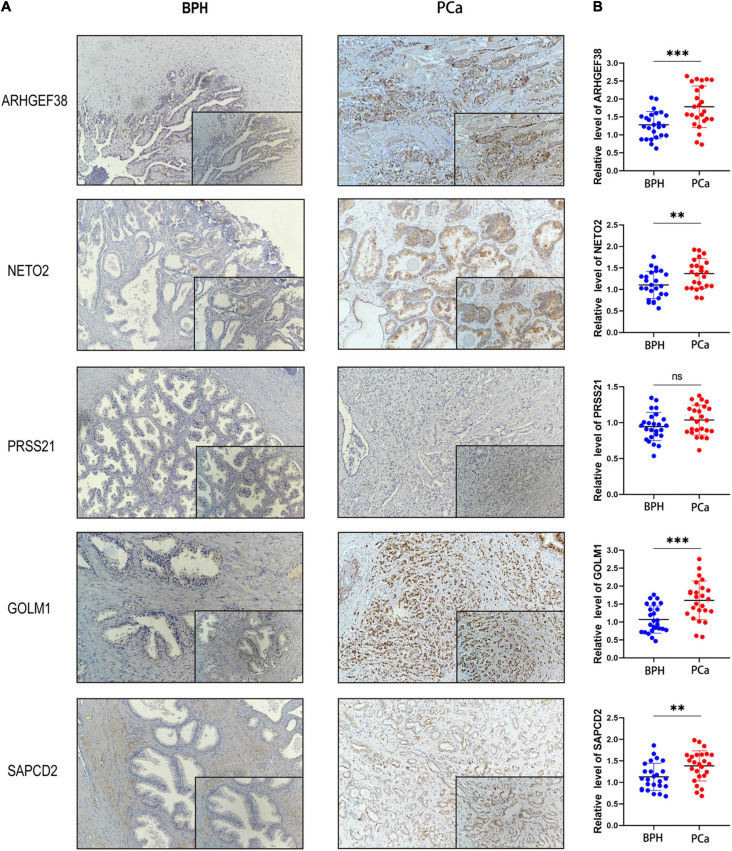
Protein and mRNA levels of signature-related genes in clinical patients. The protein levels **(A)** and mRNA levels **(B)** of ARHGEF38, NETO2, GOLM1, and SAPCD2 were higher in tumor tissues than in BPH tissues, but the levels of PRSS21 were not different in the tumor and BPH tissues. ^∗∗^*P* < 0.01, ^∗∗∗^*P* < 0.001, ns, no significance.

To validate the results of the previous comprehensive analysis, we first detected the expression levels of ARHGEF38, NETO2, PRSS21, GOLM1, and SAPCD2 in 25 paired tissue samples. As expected, the protein levels of the four risk genes (ARHGEF38, NETO2, GOLM1, and SAPCD2) were significantly higher in PCa tissues than in BPH tissues (Figure 8A), which agreed with our previous findings. However, PRSS21 protein expression was not detected in either BPH or PCa tissues. We also detected the mRNA levels of five signature-related genes by qRT–PCR, and similar results were obtained (Figure 8B). We then validated the biofunctions of ARHGEF38, NETO2, GOLM1, and SAPCD2 in PCa cells through *in vitro* experiments.

### Silencing ARHGEF38, NETO2, GOLM1, and SAPCD2 Suppresses PCa Cell Proliferation, Migration, and Invasion

**FIGURE 9:**
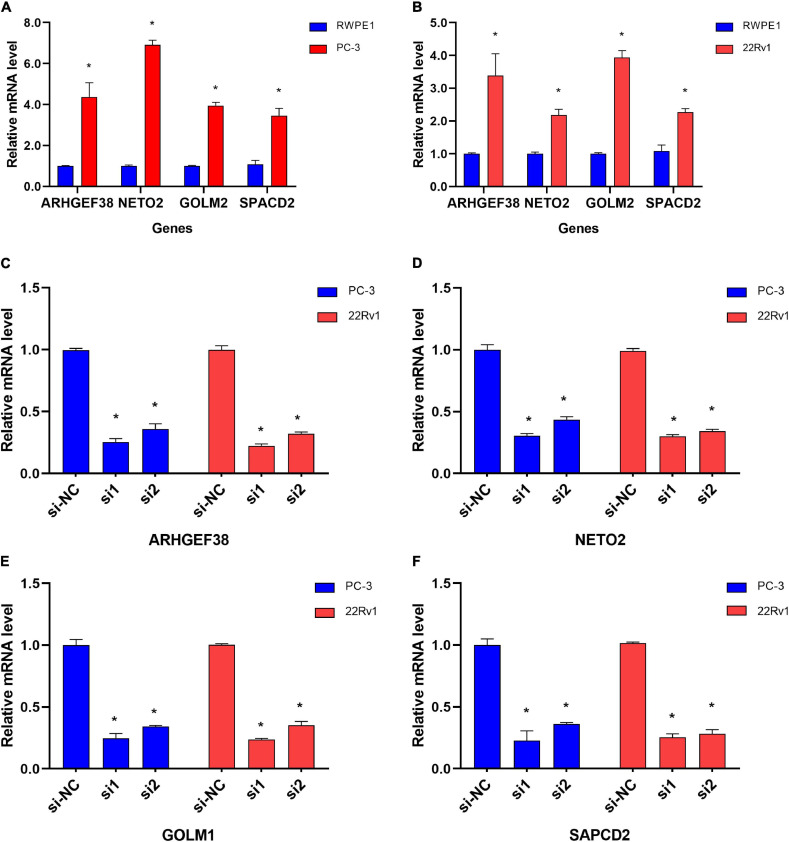
Differential mRNA expression of ARHGEF38, NETO2, GOLM1, and SAPCD2 in different cell lines. **(A,B)** ARHGEF38, NETO2, GOLM1, and SAPCD2 were significantly higher in PC-3 **(A)** and 22Rv1 **(B)** cells compared to RWPE-1 cells. **(C–F)** siRNAs inhibited the mRNA expression of ARHGEF38, NETO2, GOLM1, and SAPCD2. *P < 0.05.

To further evaluate the roles of ARHGEF38, NETO2, GOLM1, and SAPCD2 in PCa, preliminary experiments were performed *in vitro*. We first quantitatively determined the mRNA expression levels of four genes in the RWPE1 cell line and two PCa cell lines (22Rv1 and PC-3), and we found that the mRNA levels of these genes were highly expressed in both PCa cell lines compared to the RWPE1 cell line (Figures 9A,B).

**FIGURE 10:**
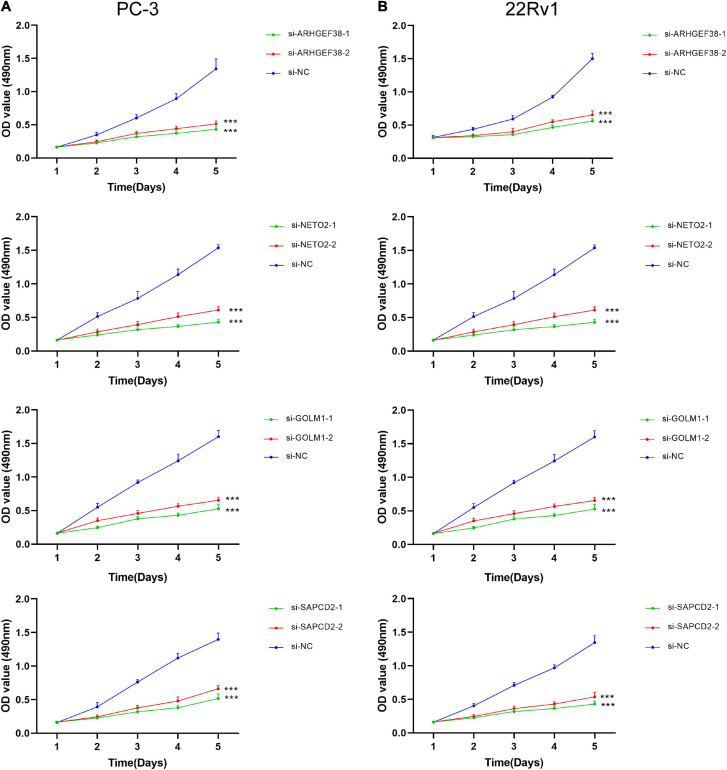
ARHGEF38, NETO2, GOLM1, and SAPCD2 promote the proliferation of PCa cells according to a MTT assay. **(A,B)** Silencing of ARHGEF38, NETO2, GOLM1, and SAPCD2 suppressed the proliferation of PC-3 cells **(A)** and 22Rv1 cells **(B)**. NC, negative control. ***P < 0.001.

**FIGURE 11:**
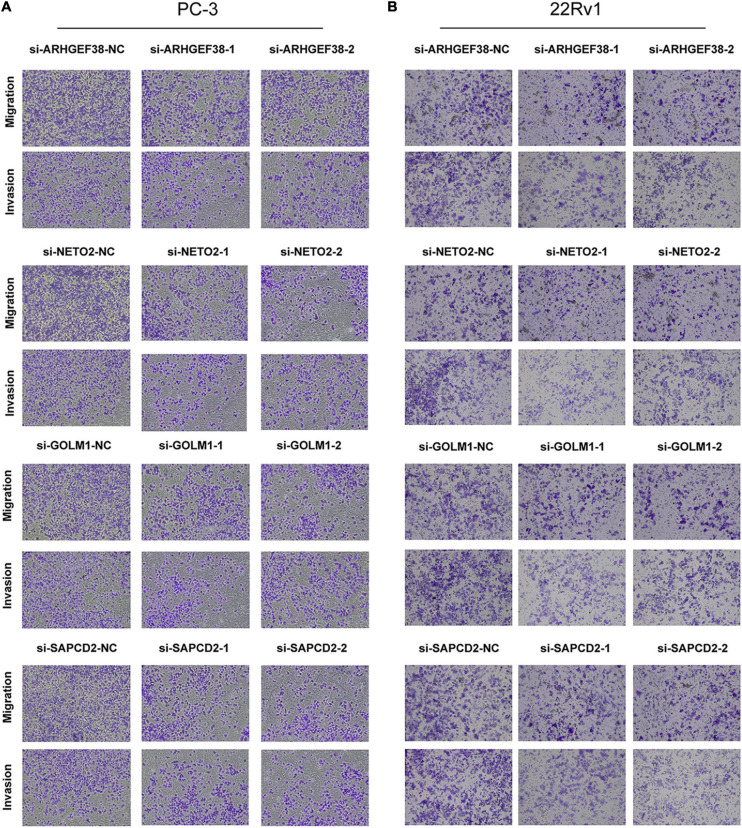
Effects of ARHGEF38, NETO2, GOLM1, and SAPCD2 on the migration and invasive abilities of PCa cells as assessed by Transwell assays. **(A)** Knockdown of ARHGEF38, NETO2, GOLM1, and SAPCD2 inhibited the migration and invasion abilities of PC-3 cells **(A)** and 22Rv1 cells **(B)**. NC, negative control.

To further examine the effects of the expression of the four genes in PCa cells, we synthesized siRNAs targeting these genes to downregulate their expression in 22Rv1 and PC-3 cells. qRT–PCR analysis showed that the mRNA expression of these genes in siRNA-transfected PCa cells was significantly downregulated compared to cells treated with negative control siRNA (si-NC) (Figures 9C–F). MTT assays were performed to evaluate the relationship between these four genes and cell proliferation. Silencing ARHGEF38, NETO2, GOLM1, and SAPCD2 inhibited the proliferative abilities of 22Rv1 (Figure 10A) and PC-3 cells (Figure 10B). In addition, Transwell migration and invasion assays demonstrated that silencing these four genes inhibited the migratory and invasion abilities of 22Rv1 (Figure 11A) and PC-3 (Figure 11B) cells compared to those transfected with si-NC.

## Discussion

Prostate cancer is biologically and clinically a heterogeneous disease whose risk varies with host and tumor characteristics, and its pathogenesis remains unclear (Tosoian et al., 2015). Despite advancements in PCa treatment modalities, treatment options for advanced modalities are still rather limited. Early diagnosis and treatment can significantly improve patients’ prognoses. However, current diagnostic methods, such as PSA assays, are defective and may result in many false positives in non-malignant cases. Thus, searching for novel tumor markers for diagnosis or prognosis prediction is of great significance.

With the rapid development of genome-sequencing technology, an increasing number of promising biomarkers have been identified to have potential value in diagnosis or prognosis prediction. Although some genes or prognostic models have been identified to predict the outcome for PCa patients by screening out DEGs, few of them have obtained differentially co-expressed genes through combining WGCNA findings with differential gene expression analysis. WGCNA is an effective method for the identification of potent genes and the underlying related biological processes in large-scale cancer gene expression profiles (Lu et al., 2014). WGCNA has been used to determine gene co-expression modules related to clinical features using a soft-threshold algorithm, allowing the co-expression network to align with the characteristics of biological networks, resulting in higher reliability and biological implications (Langfelder and Horvath, 2008).

In the present study, we first screened DEGs between PCa and normal control tissues in TCGA dataset and the GSE46602 dataset. Subsequently, we identified significant modules through WGCNA and observed that the black module in TCGA dataset and the turquoise module in the GSE46602 dataset were highly associated with PCa. After creating an intersection, we obtained 124 overlapping genes that possessed relevant expression patterns by combining WGCNA findings with DEG analysis.

Gene Ontology enrichment analysis showed that 124 overlapping genes were enriched in several biological terms, including gland development, epithelial cell proliferation, and positive regulation of MAP kinase activity, confirming their involvement in the development of PCa (Yu et al., 2007; Mayo et al., 2017; Madueke et al., 2019). The results from KEGG pathway analysis indicated that the PI3K-Akt signaling pathway was the most significantly identified pathway. It has been reported that the PI3K-Akt signaling pathway regulates survival, proliferation, growth, migration, and angiogenesis (Sarker et al., 2009). Activation of the PI3K/Akt pathway is vital to PCa metastasis and drug resistance to chemotherapy (Toren and Zoubeidi, 2014). Furthermore, the KEGG pathways were also enriched in the Hippo signaling pathway and Wnt signaling pathway, which have been demonstrated to play important roles in different stages of PCa initiation, progression, and regulation of AR signaling (Murillo-Garzón and Kypta, 2017; Salem and Hansen, 2019). Together, these results suggested that these overlapping genes are involved in the occurrence and progression of PCa.

Based on the overlapping genes, we then used Cox regression analysis to construct a five-gene prognostic signature to predict the outcome of PCa in TCGA dataset. The patients in the high-risk group had a significantly shorter survival time than those in the low-risk group. We then further validated the effectiveness of the signature in predicting BCR-free survival in the GSE46602 dataset. ROC analysis demonstrated that the constructed prognostic signature might be better than the other clinical parameters and more accurate in differentiating PCa patients. Further Cox regression analysis indicated that the signature was an independent prognostic indicator for the prognostic assessment of PCa. In clinical situations, we suggest more frequent follow-up and active treatment in PCa patients with higher scores to greatly improve prognosis, corresponding to the concept of precision medicine.

These five genes (ARHGEF38, NETO2, PRSS21, GOLM1, and SAPCD2) in our signature have been previously reported to participate in tumorigenesis and progression. Rho guanine nucleotide exchange Factor 38 (ARHGEF38), a member of the Rho guanine nucleotide exchange factor family, may have important significance in regulating membrane protrusions to guide cell migration (Liu et al., 2020). Liu et al. (2020) found that ARHGEF38 is significantly overexpressed in PCa compared to benign prostate hyperplasia, especially in high-grade prostate cancer. In addition, compared to tissues without lymph node metastasis, ARHGEF38 protein presents a much higher expression in PCa tissues with lymph node metastasis. These results suggest that ARHGEF38 may promote PCa migration and metastasis, thereby contribute to PCa progression (Liu et al., 2020). Our results confirmed that ARHGEF38 was upregulated in PCa and that it may serve as an oncogene, thereby agreeing with previously reported studies.

Neuropilin and tolloid-like 2 (NETO2), a member of the subfamily containing CUB and LDLa domains, has recently been reported to be upregulated in different types of solid tumors, such as colorectal cancer (Hu et al., 2015), pancreatic cancer (Li et al., 2019), and hepatocellular carcinoma (Villa et al., 2016). A recent study of gastric cancer (Liu et al., 2019) has demonstrated that NETO2 is a vital oncoprotein that activates the PI3K/Akt/NF-κB/Snail axis to contribute to gastric carcinoma invasion and metastasis by inducing EMT. Interestingly, our results indicated that the PI3K-Akt signaling pathway was the most significantly identified pathway in the KEGG pathway analysis. Activation of the PI3K/Akt pathway was highly correlated with PCa progression. Based on these results, we speculate that NETO2 may enhance PCa progression through the PI3K/Akt pathway. However, further experiments are needed to confirm this hypothesis.

Serine protease 21 (PRSS21), also known as testisin, encodes a predicted glycosyl-phosphatidylinositol–linked (GPI)-linked or membrane-anchored protein (Hooper et al., 1999). PRSS21 has been reported to be upregulated in premeiotic testicular germ cells but not in other normal adult tissues (Hooper et al., 1999). Tang et al. (2005) found that PRSS21 mRNA is overexpressed in ovarian tumors but has restricted expression in normal human tissues other than the testis. However, Conway et al. (2019) unexpectedly discovered that increased PRSS21 activity has a minor impact on cell proliferation but inhibits intraperitoneal tumor metastasis, resulting in a significantly reduced tumor burden. In our research, we found that the expression of PRSS21 was higher in the normal samples than in the PCa samples in TCGA-PCa dataset. Nevertheless, PRSS21 was rarely or never expressed in both PCa and normal control tissues in our experimental validation, which agreed with previous studies. However, the role of PRSS21 in prostate cancer needs further exploration.

Golgi membrane protein 1 (GOLM1), a transmembrane glycoprotein of the Golgi cisternae, is involved in the carcinogenesis of many types of cancers. Yan et al. (2018) reported that GOLM1 promotes proliferation, migration, and invasion but inhibits apoptosis in PCa cell lines (DU145, PC-3, and 22Rv1) by activating the PI3K-Akt-mTOR signaling pathway, supporting our hypothesis that NETO2 may promote the progression of PCa by upregulating the PI3K/Akt pathway. In addition, GOLM1 facilitates hepatocellular carcinoma metastasis by regulating membrane protein trafficking, especially the signaling kinetics of EGFR/RTK complex recycling (Ye et al., 2016).

Suppressor anaphase-promoting complex domain containing 2 (SAPCD2), a cell cycle-dependent gene, has been reported to be upregulated in many solid tumors. For example, SAPCD2 enhances breast cancer cell proliferation ability by modulating the expression of YAP/TAZ, thereby promoting the progression of breast cancer. SAPCD2 may be associated with gastric cancer cell proliferation. Silencing SAPCD2 significantly inhibits cancer cell proliferation and colony formation of gastric cancer cells. The significance of SAPCD2 in PCa is currently unclear and requires further investigation to elucidate the mechanisms that contribute to PCa progression.

To further investigate the cause of abnormal expression of the signature-related genes in PCa, we used DiseaseMeth 2.0 and MEXPRESS to explore the relationship between DNA methylation patterns and gene expression levels. Compared to normal tissues, ARHGEF38, NETO2, GOLM1, and SAPCD2 were hypomethylated in prostate tumors, while PRSS21 was hypermethylated in prostate tumors compared, which may be the underlying mechanism for the abnormal expression of the five genes in PCa. In addition, our analysis suggested that genetic alterations might contribute to aberrant gene expression to some extent.

The present study demonstrated that silencing ARHGEF38 and GOLM1 attenuated PCa cell proliferation, migration, and invasion abilities, which agreed with previous research (Yan et al., 2018; Liu et al., 2020). Although NETO2 and SAPCD2 have been reported to be involved in angiogenesis in different tumor types, the potential roles of these two genes in PCa were not elucidated. Through a series of functional analyses (*in vitro*), we verified the pro-cancer abilities of NETO2 and SAPCD2, which provided a new approach for further study on the mechanism in PCa.

Although the results of our study are encouraging, we should also recognize several limitations of our work. First, larger sample clinical studies are needed to validate the clinical significance of the constructed signature as an independent prognostic indicator for PCa, which we will concentrate on in future research. Second, no detailed systematic therapy data were integrated into the model, which might further improve the performance.

## Conclusion

Our study constructed a prognostic signature for PCa patients by combining WGCNA with DEG analysis. The five identified signature-related genes were significantly associated with the progression and prognosis of PCa. Moreover, ARHGEF38, NETO2, GOLM1, and SAPCD2 promoted the proliferation, migration, and invasion of PCa. This research not only discovered possible candidate markers and targets for PCa diagnosis or treatment but also provided a better understanding of the potential etiology and molecular pathogenesis of PCa by integrating multiple bioinformatics approaches and validating experiments.

## Data Availability Statement

Publicly available datasets were analyzed in this study. This data can be found here: https://portal.gdc.cancer.gov/ and https://www.ncbi.nlm.nih.gov.

## Ethics Statement

The studies involving human participants were reviewed and approved by the Ethical Committee of Third Affiliated Hospital of Sun Yat-sen University. The patients/participants provided their written informed consent to participate in this study.

## Author Contributions

ZS and YM conceived, designed, and wrote the manuscript. XZ, SL, and HW assisted in specimen collection and performed the experimental work. CZ, CX, YC, and YQ performed the data analysis and generated the figures. YW and KL helped with the manuscript and data review. All authors contributed to the article and approved the submitted version.

## Conflict of Interest

The authors declare that the research was conducted in the absence of any commercial or financial relationships that could be construed as a potential conflict of interest.

## Publisher’s Note

All claims expressed in this article are solely those of the authors and do not necessarily represent those of their affiliated organizations, or those of the publisher, the editors and the reviewers. Any product that may be evaluated in this article, or claim that may be made by its manufacturer, is not guaranteed or endorsed by the publisher.

## Footnotes

1
https://portal.gdc.cancer.gov/

2
https://www.gencodegenes.org/

3
https://www.ncbi.nlm.nih.gov/

4
https://www.oncomine.org/resource/main.htm

5
http://cbioportal.org

6
http://gepia.cancer-pku.cn/

7
http://bio-bigdata.hrbmu.edu.cn/diseasemeth/index.html

8
https://mexpress.be/

## References

[B1] Benjamini Y. Hochberg Y. (1995). Controlling the false discovery rate: a practical and powerful approach to multiple testing. *J. R. Stat. Soc.* 57 289–300. 10.1111/j.2517-6161.1995.tb02031.x

[B2] Botía J. A. Vandrovcova J. Forabosco P. Guelfi S. D’Sa K. Hardy J. (2017). An additional k-means clustering step improves the biological features of WGCNA gene co-expression networks. *BMC Syst. Biol.* 11:47. 10.1186/s12918-017-0420-6 28403906 PMC5389000

[B3] Brockman J. A. Alanee S. Vickers A. J. Scardino P. T. Wood D. P. Kibel A. S. (2015). Nomogram predicting prostate cancer-specific mortality for men with biochemical recurrence after radical prostatectomy. *Eur. Urol.* 67 1160–1167. 10.1016/j.eururo.2014.09.019 25301759 PMC4779062

[B4] Cerami E. Gao J. Dogrusoz U. Gross B. E. Sumer S. O. Aksoy B. A. (2012). The cBio cancer genomics portal: an open platform for exploring multidimensional cancer genomics data. *Cancer Discov.* 2 401–404. 10.1158/2159-8290.cd-12-0095 22588877 PMC3956037

[B5] Conway G. D. Buzza M. S. Martin E. W. Duru N. Johnson T. A. Peroutka R. J. (2019). PRSS21/testisin inhibits ovarian tumor metastasis and antagonizes proangiogenic angiopoietins ANG2 and ANGPTL4. *J. Mol. Med.* 97 691–709. 10.1007/s00109-019-01763-3 30911775 PMC6513752

[B6] Ferrer-Batallé M. Llop E. Ramírez M. Aleixandre R. N. Saez M. Comet J. (2017). Comparative study of blood-based biomarkers, α2,3-Sialic Acid PSA and PHI, for high-risk prostate cancer detection. *Int. J. Mol. Sci.* 18:845. 10.3390/ijms18040845 28420168 PMC5412429

[B7] Grossman D. C. Curry S. J. Owens D. K. Bibbins-Domingo K. Caughey A. B. Davidson K. W. (2018). Screening for prostate cancer: US preventive services task force recommendation statement. *Jama* 319 1901–1913. 10.1001/jama.2018.3710 29801017

[B8] Hooper J. D. Nicol D. L. Dickinson J. L. Eyre H. J. Scarman A. L. Normyle J. F. (1999). Testisin, a new human serine proteinase expressed by premeiotic testicular germ cells and lost in testicular germ cell tumors. *Cancer Res.* 59 3199–3205. 10397266

[B9] Hu L. Chen H. Y. Cai J. Yang G. Z. Feng D. Zhai Y. X. (2015). Upregulation of NETO2 expression correlates with tumor progression and poor prognosis in colorectal carcinoma. *BMC Cancer* 15:1006. 10.1186/s12885-015-2018-y 26699544 PMC4690429

[B10] Koch A. Jeschke J. Van Criekinge W. van Engeland M. De Meyer T. (2019). MEXPRESS update 2019. *Nucleic Acids Res.* 47 W561–W565. 10.1093/nar/gkz445 31114869 PMC6602516

[B11] Kohaar I. Petrovics G. Srivastava S. (2019). A rich array of prostate cancer molecular biomarkers: opportunities and challenges. *Int. J. Mol. Sci.* 20:1813. 10.3390/ijms20081813 31013716 PMC6515282

[B12] Langfelder P. Horvath S. (2008). WGCNA: an R package for weighted correlation network analysis. *BMC Bioinformatics* 9:559. 10.1186/1471-2105-9-559 19114008 PMC2631488

[B13] Li Y. Zhang Y. Liu J. (2019). NETO2 promotes pancreatic cancer cell proliferation, invasion and migration via activation of the STAT3 signaling pathway. *Cancer Manag. Res.* 11 5147–5156. 10.2147/cmar.s204260 31239769 PMC6560188

[B14] Liu J. Y. Jiang L. He T. Liu J. J. Fan J. Y. Xu X. H. (2019). NETO2 promotes invasion and metastasis of gastric cancer cells via activation of PI3K/Akt/NF-κB/Snail axis and predicts outcome of the patients. *Cell Death Dis.* 10:162. 10.1038/s41419-019-1388-5 30770791 PMC6377647

[B15] Liu K. Wang A. Ran L. Zhang W. Jing S. Wang Y. (2020). ARHGEF38 as a novel biomarker to predict aggressive prostate cancer. *Genes Dis.* 7 217–224. 10.1016/j.gendis.2019.03.004 32215291 PMC7083745

[B16] Loeb S. Bjurlin M. A. Nicholson J. Tammela T. L. Penson D. F. Carter H. B. (2014). Overdiagnosis and overtreatment of prostate cancer. *Eur. Urol.* 65 1046–1055. 10.1016/j.eururo.2013.12.062 24439788 PMC4113338

[B17] Lu X. Deng Y. Huang L. Feng B. Liao B. (2014). A co-expression modules based gene selection for cancer recognition. *J. Theor. Biol.* 362 75–82. 10.1016/j.jtbi.2014.01.005 24440175

[B18] Madueke I. Hu W. Y. Hu D. Swanson S. M. Vander Griend D. Abern M. (2019). The role of WNT10B in normal prostate gland development and prostate cancer. *Prostate* 79 1692–1704. 10.1002/pros.23894 31433503 PMC9639854

[B19] Mayo J. C. Hevia D. Quiros-Gonzalez I. Rodriguez-Garcia A. Gonzalez-Menendez P. Cepas V. (2017). IGFBP3 and MAPK/ERK signaling mediates melatonin-induced antitumor activity in prostate cancer. *J. Pineal Res.* 62:1188–1189. 10.1111/jpi.12373 27736013

[B20] Mortensen M. M. Høyer S. Lynnerup A. S. Ørntoft T. F. Sørensen K. D. Borre M. (2015). Expression profiling of prostate cancer tissue delineates genes associated with recurrence after prostatectomy. *Sci. Rep.* 5:16018. 10.1038/srep16018 26522007 PMC4629186

[B21] Murillo-Garzón V. Kypta R. (2017). WNT signalling in prostate cancer. *Nat. Rev. Urol.* 14 683–696. 10.1038/nrurol.2017.144 28895566

[B22] Rhodes D. R. Yu J. Shanker K. Deshpande N. Varambally R. Ghosh D. (2004). ONCOMINE: a cancer microarray database and integrated data-mining platform. *Neoplasia* 6 1–6. 10.1016/s1476-5586(04)80047-2 15068665 PMC1635162

[B23] Ritchie M. E. Phipson B. Wu D. Hu Y. Law C. W. Shi W. (2015). limma powers differential expression analyses for RNA-sequencing and microarray studies. *Nucleic Acids Res.* 43:e47. 10.1093/nar/gkv007 25605792 PMC4402510

[B24] Rui X. Shao S. Wang L. Leng J. (2019). Identification of recurrence marker associated with immune infiltration in prostate cancer with radical resection and build prognostic nomogram. *BMC Cancer* 19:1179. 10.1186/s12885-019-6391-9 31795990 PMC6892211

[B25] Saad F. (2019). Quality of life in men with prostate cancer. *Lancet Oncol.* 20 325–326. 10.1016/s1470-2045(18)30863-5 30713037

[B26] Salem O. Hansen C. G. (2019). The hippo pathway in prostate cancer. *Cells* 8:370. 10.3390/cells8040370 31018586 PMC6523349

[B27] Sarker D. Reid A. H. Yap T. A. de Bono J. S. (2009). Targeting the PI3K/AKT pathway for the treatment of prostate cancer. *Clin. Cancer Res.* 15 4799–4805. 10.1158/1078-0432.ccr-08-0125 19638457

[B28] Shao N. Tang H. Mi Y. Zhu Y. Wan F. Ye D. (2020). A novel gene signature to predict immune infiltration and outcome in patients with prostate cancer. *Oncoimmunology* 9:1762473. 10.1080/2162402x.2020.1762473 32923125 PMC7458664

[B29] Siegel R. L. Miller K. D. Jemal A. (2020). Cancer statistics, 2020. *CA Cancer J. Clin.* 70 7–30. 10.3322/caac.21590 31912902

[B30] Skolarus T. A. Wolf A. M. Erb N. L. Brooks D. D. Rivers B. M. Underwood W. III (2014). American Cancer Society prostate cancer survivorship care guidelines. *CA Cancer J. Clin.* 64 225–249. 10.3322/caac.21234 24916760

[B31] Tang T. Kmet M. Corral L. Vartanian S. Tobler A. Papkoff J. (2005). Testisin, a glycosyl-phosphatidylinositol-linked serine protease, promotes malignant transformation in vitro and in vivo. *Cancer Res.* 65 868–878. 15705885

[B32] Tang Z. Li C. Kang B. Gao G. Li C. Zhang Z. (2017). GEPIA: a web server for cancer and normal gene expression profiling and interactive analyses. *Nucleic Acids Res.* 45 W98–W102. 10.1093/nar/gkx247 28407145 PMC5570223

[B33] To K. Y. (2000). Identification of differential gene expression by high throughput analysis. *Comb. Chem. High Throughput Screen* 3 235–241. 10.2174/1386207003331616 10903382

[B34] Toren P. Zoubeidi A. (2014). Targeting the PI3K/Akt pathway in prostate cancer: challenges and opportunities (review). *Int. J. Oncol.* 45 1793–1801. 10.3892/ijo.2014.2601 25120209

[B35] Tosoian J. J. Mamawala M. Epstein J. I. Landis P. Wolf S. Trock B. J. (2015). Intermediate and longer-term outcomes from a prospective active-surveillance program for favorable-risk prostate cancer. *J. Clin. Oncol.* 33 3379–3385. 10.1200/jco.2015.62.5764 26324359 PMC4863946

[B36] Villa E. Critelli R. Lei B. Marzocchi G. Cammà C. Giannelli G. (2016). Neoangiogenesis-related genes are hallmarks of fast-growing hepatocellular carcinomas and worst survival. Results from a prospective study. *Gut* 65 861–869. 10.1136/gutjnl-2014-308483 25666192

[B37] Xiong Y. Wei Y. Gu Y. Zhang S. Lyu J. Zhang B. (2017). DiseaseMeth version 2.0: a major expansion and update of the human disease methylation database. *Nucleic Acids Res.* 45 D888–D895. 10.1093/nar/gkw1123 27899673 PMC5210584

[B38] Yan G. Ru Y. Wu K. Yan F. Wang Q. Wang J. (2018). GOLM1 promotes prostate cancer progression through activating PI3K-AKT-mTOR signaling. *Prostate* 78 166–177. 10.1002/pros.23461 29181846

[B39] Ye Q. H. Zhu W. W. Zhang J. B. Qin Y. Lu M. Lin G. L. (2016). GOLM1 modulates EGFR/RTK cell-surface recycling to drive hepatocellular carcinoma metastasis. *Cancer Cell* 30 444–458. 10.1016/j.ccell.2016.07.017 27569582 PMC5021625

[B40] Yu S. Wang X. Ng C. F. Chen S. Chan F. L. (2007). ERRgamma suppresses cell proliferation and tumor growth of androgen-sensitive and androgen-insensitive prostate cancer cells and its implication as a therapeutic target for prostate cancer. *Cancer Res.* 67 4904–4914. 10.1158/0008-5472.can-06-3855 17510420

[B41] Zeng X. Sanalkumar R. Bresnick E. H. Li H. Chang Q. Keleş S. (2013). jMOSAiCS: joint analysis of multiple ChIP-seq datasets. *Genome Biol.* 14:R38. 10.1186/gb-2013-14-4-r38 23844871 PMC4053760

