# Supplementary material for: Identification of ARHGEF38, NETO2, GOLM1, and SAPCD2 Associated With Prostate Cancer Progression by Bioinformatic Analysis and Experimental Validation
Source: Front Cell Dev Biol. 2021 Sep 1;9:718638. doi: 10.3389/fcell.2021.718638 (PMC8440839; doi:10.3389/fcell.2021.718638)
Supplement: Supplementary file 1 [file Data_Sheet_1.DOCX]

**Supplementary Methods**

**Supplementary Tables**

**Supplementary Table 1. Primer sequences used for qRT-PCR in this study.**

| Gene Name | Forward Primer | Reverse Primer |
| --- | --- | --- |
| ARHGEF38 | 5'-TAGCTGAGACCTTAACCCCAG-3' | 5'-CACATCCAGCCTATCAGTCTTTT-3' |
| NETO2 | 5'-GCTGATGGAATAGTGCGCTCT-3' | 5'-CAGATGCAATCAACGGCTTGG-3' |
| PRSS21 | 5'-GCGCCGTTATCAGGACCAT-3' | 5'-CTCCGCATACGTGGGAATCC-3' |
| GOLM1 | 5'-AGAGCGTCAACAAGCTGTACC-3' | 5'-CAGCCTGCCGTAATTCCTCTG-3' |
| SAPCD2 | 5'-TGCCCAAGGTACAAGAGGTG-3' | 5'-CTGGGTGAGGAGTCGGTTCT-3' |
| β-actin | 5′-TTGCGTTACACCCTTTCTTG-3′ | 5′-CACCTTCACCGTTCCAGTTT-3′ |

**Supplementary Table 2. The oligonucleotides used in this study.**

| Gene Name | Target Sequence |
| --- | --- |
| si-ARHGEF38-1 | 5'-AAGGATTATCTCAATGATCTA-3' |
| si-ARHGEF38-2 | 5'-TGCAAGTAATTGGAGAAGTAT-3' |
| si-NETO2-1 | 5'-TCCTCAAAGTGTTGTTAATAA-3' |
| si-NETO2-2  si-GOLM1-1  si-GOLM1-2  si-SAPCD2-1  si-SAPCD2-2  si-NC | 5’-ATCGTTACTGTGGCGTGAAAA-3’  5′-CAGGGAAUGACAGAAACAUTT-3′  5′-GUGCUUGGUAACAGCAAGUTT-3′  5′-CACAAGGAAACAATACACTAT-3′  5′-ACCCAGATACTAGAATTATCA-3′  5′-GCUCAACCGUGAAGUUAUAUU-3′ |

**Supplementary Figures**


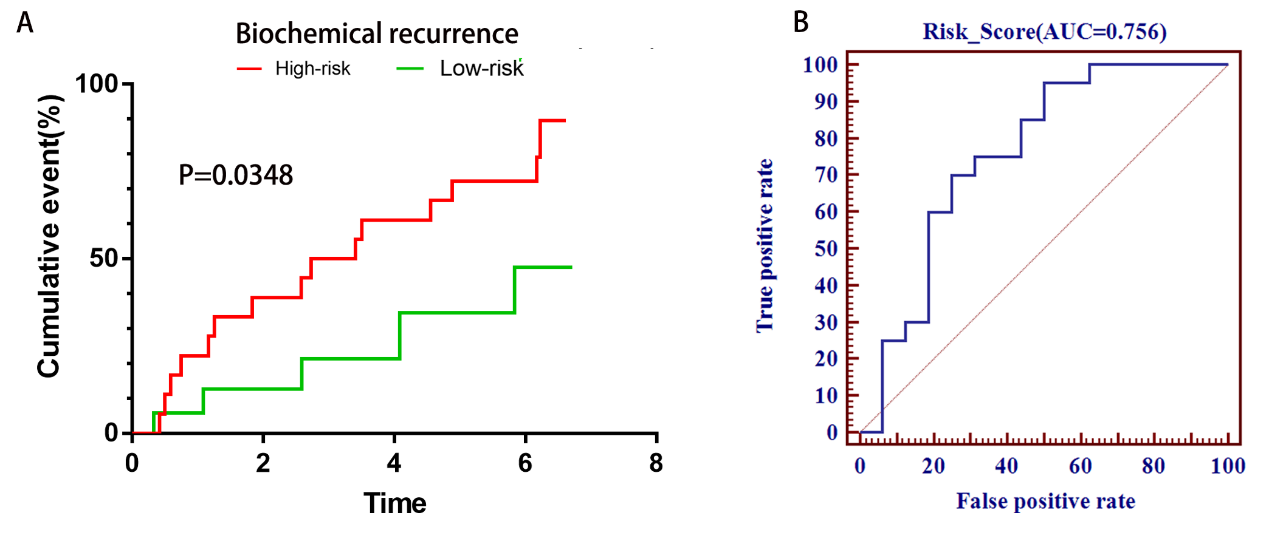


**Supplementary Figure 1. Kaplan‑Meier analysis for biochemical recurrence-free survival in the GSE46602 dataset**. (A) The K-M curves for BCR-free survival in the high- and low-risk groups; (B) The ROC analysis showed great classifying efficacy.


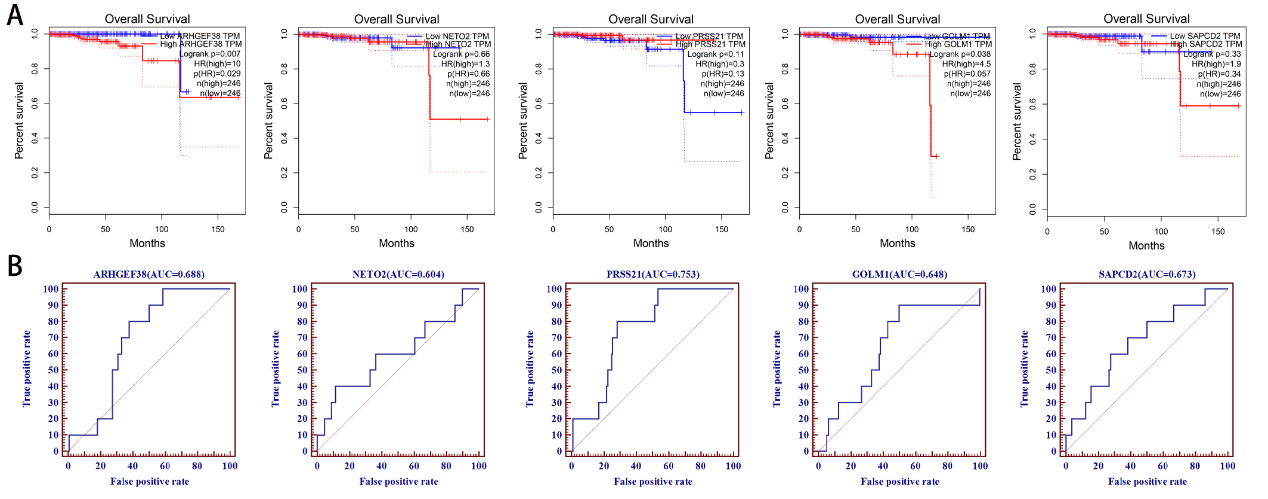


**Supplementary Figure 2. Overall survival (OS) analysis and receiver operating characteristic (ROC) analysis of the five signature-related genes.** (A) OS analysis of ARHGEF38, NETO2, PRSS21, GOLM1, and SAPCD2 in TCGA‐PCa patients based on the GEPIA2 database. (B) ROC analysis of ARHGEF38, NETO2, PRSS21, GOLM1, and SAPCD2 in TCGA‐PCa patients.


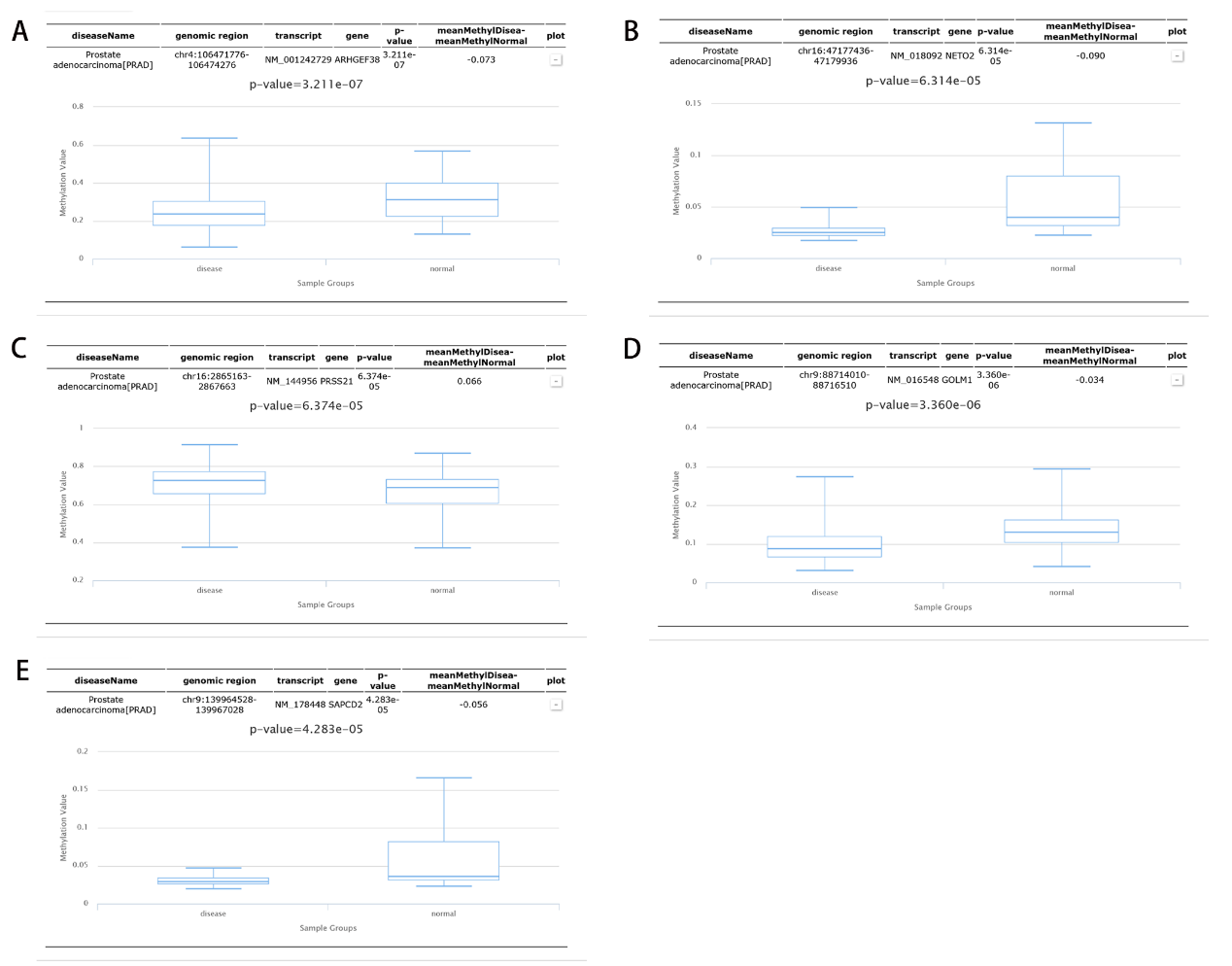


**Supplementary Figure 3. Methylation** **analyses of the five signature-related genes in PCa tissues and normal prostate tissue based on DiseaseMeth 2.0.** (A)ARHGEF38, (B) NETO2, (C) PRSS21, (D) GOLM1, and (E) SAPCD2.


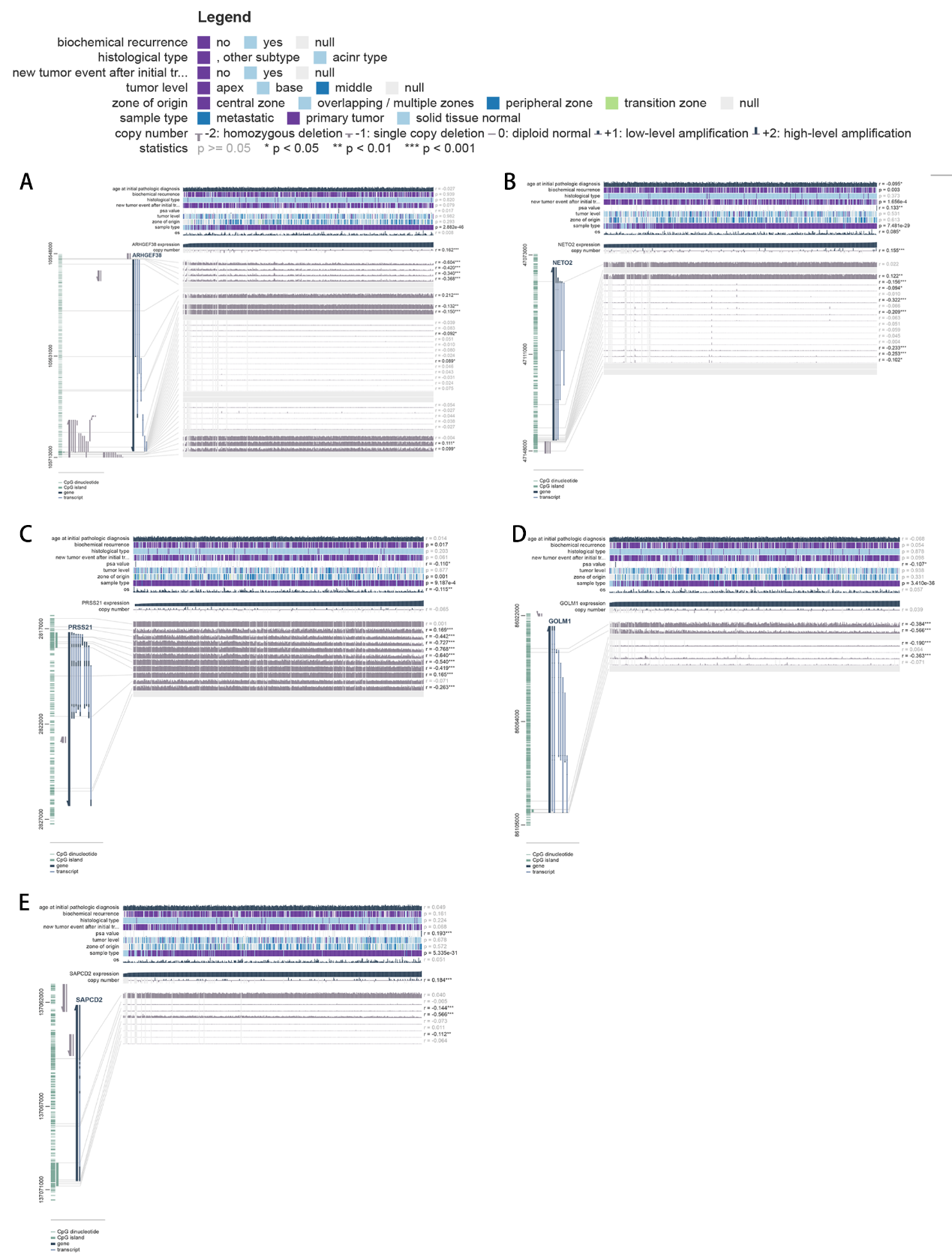


**Supplementary Figure 4. Association of Methylation sites with expression of the five signature-related genes.** The methylation sites of (A) ARHGEF38, (B) NETO2, (C) PRSS21, (D) GOLM1, and (E) SAPCD2 DNA sequences, and their associations with gene expression, were visualized using MEXPRESS database. The legend shows the different values are for the categorical variables. The expression of query genes is illustrated by the navy blue line in the center of the plot. The samples are sorted by the expression (from low to high) of the gene that was entered. Each row below it shows the DNA methylation data for a single probe on the Infinium microarray. These are the *P* values and correlation coefficients for methylation sites and query gene expression on the right side.
